# Regulated cell death in hypoxic-ischaemic encephalopathy: recent development and mechanistic overview

**DOI:** 10.1038/s41420-024-02014-2

**Published:** 2024-06-11

**Authors:** Lingzhi Wu, Enqiang Chang, Hailin Zhao, Daqing Ma

**Affiliations:** 1 Division of Anaesthetics, Pain Medicine and Intensive Care, Department of Surgery and Cancer, Faculty of Medicine, Imperial College London, Chelsea and Westminster Hospital, London, UK; 2 Perioperative and Systems Medicine Laboratory, The Children’s Hospital, Zhejiang University School of Medicine, National Clinical Research Center for Child Health, Hangzhou, 310052 China

**Keywords:** Trauma, Experimental models of disease

## Abstract

Hypoxic-ischaemic encephalopathy (HIE) in termed infants remains a significant cause of morbidity and mortality worldwide despite the introduction of therapeutic hypothermia. Depending on the cell type, cellular context, metabolic predisposition and insult severity, cell death in the injured immature brain can be highly heterogenous. A continuum of cell death exists in the H/I-injured immature brain. Aside from apoptosis, emerging evidence supports the pathological activation of necroptosis, pyroptosis and ferroptosis as alternative regulated cell death (RCD) in HIE to trigger neuroinflammation and metabolic disturbances in addition to cell loss. Upregulation of autophagy and mitophagy in HIE represents an intrinsic neuroprotective strategy. Molecular crosstalk between RCD pathways implies one RCD mechanism may compensate for the loss of function of another. Moreover, mitochondrion was identified as the signalling “hub” where different RCD pathways converge. The highly-orchestrated nature of RCD makes them promising therapeutic targets. Better understanding of RCD mechanisms and crosstalk between RCD subtypes likely shed light on novel therapy development for HIE. The identification of a potential RCD converging node may open up the opportunity for simultaneous and synergistic inhibition of cell death in the immature brain.

## Facts

- In hypoxic-ischaemic encephalopathy (HIE), the immature brain displays a “continuum” of cell death phenotypes that are uniquely regulated
- Aside from apoptosis, hypoxia-ischaemia challenge activates necroptosis, pyroptosis and ferroptosis to contribute to the demise of vulnerable cell types in the neonatal brain
- Autophagy and mitophagy are upregulated in HIE and likely exert neuroprotective functions

## Open questions

- Mitochondrion may serve as the signalling hub where different RCD pathways converge to highlight opportunity for simultaneous targeting
- As our current understanding on cell death mechanisms during hypoxic-ischaemic brain injury predominantly stems from studies conducted on the adult brains, more research on the immature, developing brains are needed as their unique vulnerability, plasticity and regenerative capacity could give rise to distinct injury mechanisms
- Clinical studies and human evidences on HIE are extremely limited and there is a critical need to expand our research efforts to enhance the translatability of mechanistic insights gained from animal studies

## Introduction

Hypoxic-ischaemic encephalopathy (HIE) resulting from birth asphyxia contributes significantly to perinatal mortality and morbidity worldwide. The reported incidence of HIE ranges between 1–6 per 1000 live births in developed nations [1] and can rise to 26/1000 in developing countries [2]. 10–15% affected infants would die during the neonatal period [3] and is equal to approximately 1 million neonatal deaths annually [4]. HIE specifically refers to a scenario of neonatal encephalopathy (NE) where hypoxia-ischaemia during the perinatal period is evident [3, 5]. Moderate hypothermia has become the standard of care for termed infants ($\ge$ 36 weeks gestation) born with HIE in developed countries [2, 6], leading to significant reductions in mortality and long-term major disability [7–10] in moderate-to-severe cases of HIE. It is, however, crucial to acknowledge that a considerable proportion (~46%) of moderate-to-severe HIE treated with therapeutic hypothermia still had major adverse outcomes (mortality and/or neurodevelopmental disability) at 18 months [11].

**Fig. 1:**
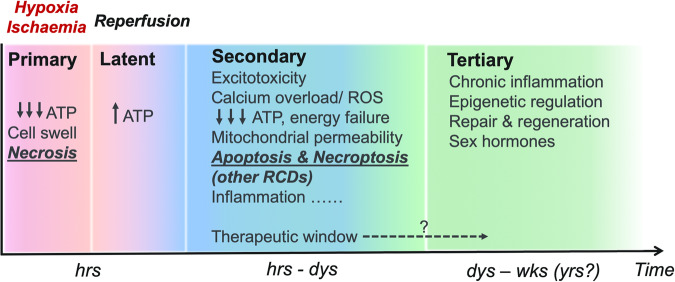
The distinct phases of hypoxic-ischaemic encephalopathy (HIE) injury in termed infants. Brain hypoxia-ischaemia (I/R) triggers an immediate, early primary injury phase that is characterised by rapid ATP decline, cell swelling and necrotic cell death. A brief latent phase ensues from reperfusion with temporary ATP recovery but is only to be followed by further episodes of injury. The secondary injury phase encompasses a chain of pathophysiological events, including excitotoxicity, calcium overload, oxidative stress, secondary energy failure and increased mitochondrial permeability through mitochondrial permeability transition pore opening (mPTP, inner mitochondrial membrane) and mitochondrial outer membrane permeabilisation (MOMP). I/R insult also initiates a neuro-inflammatory response that may persist as chronic inflammation to alter brain development and neural plasticity for years. Evidences also point to a tertiary injury phase with long-term inflammatory/anti-inflammatory modulation and epigenetic regulation on neurogenesis, differentiation and myelination, which are also subjected to the later influences of sex hormones. A clinical therapeutic window of between 6 and 24 h after initial H/I insult has been identified, depending on the neuroprotective strategies, and attempts are undergoing to further extend the therapeutic window.

The current consensus is that neonatal hypoxia-ischaemia (H/I) brain injury in the newborns unfolds in three phases [6, 12, 13] (Fig. 1). The secondary injury phase by far has received the most research attention. Apoptosis along with other more recently identified forms of regulated cell death (RCD) are believed to predominate in the secondary injury phase, and this understanding offers great hope for selective targeting of the different RCD. There is also increasing appreciation for a tertiary injury phase for HIE that features prolonged inflammation, epigenetic changes and tissue remodelling [14]. As the role of apoptosis in HIE has already been reviewed in-depth elsewhere [6, 15], the aim of the current review is to provide an updated, mechanistic overview on the alternative RCD mechanisms (namely necroptosis, pyroptosis, ferroptosis, autophagy and mitophagy) and their involvement in HIE of termed infants. Moreover, we provide a comparison of H/I-induced RCD between the adult and developing brains to highlight the overlapping mechanisms of injury. Continuous effort at elucidating the cellular injury mechanisms of HIE may pave way for novel target identification and therapeutic development to improve survival and long-term outcomes.

## Necroptosis

In hypoxia-ischaemia injured immature brain exists a “continuum”/spectrum of cell death phenotypes where classical apoptosis and classical necrosis are at the two extremities, and in between falls different hybrid forms of cell death [16]. Regulated necrosis, termed necroptosis, more closely resembles the appearance of classical necrosis, with early plasma membrane permeabilisation, swollen organelles (endoplasmic reticulum, mitochondria), translucent cytoplasm and random nuclear dissolution [15, 17]. Necroptosis is orchestrated by receptor-interacting protein kinases 1 and 3 (RIPK1/3) and their downstream pseudokinase mixed-lineage kinase-domain like (MLKL) through a chain of phosphor-activation [18, 19]. Common triggers of necroptosis include cell death receptor activation by cognate ligands (e.g. TNF-α, FasL, TNF-related apoptosis-inducing ligand – TRAIL), viral infection (activation of toll-like receptors 3 and 4) and ischaemic injury in vivo.

### Necroptosis in brain disorders

In the central nervous system (CNS), increased necroptosis activity and elevated necrosome expression are linked to oligodendrocyte vulnerability, axonal degeneration or neuronal loss in chronic neuroinflammatory and neurodegenerative conditions. Upregulated expression of RIPK1, RIPK3 and MLKL and enhanced association between necrosome components have been observed in a wide range of CNS disorders with inflammatory component, including multiple sclerosis (MS) [20, 21], amyotrophic lateral sclerosis (ALS) [22], Alzheimer’s disease (AD) [23, 24] and Parkinson’s disease (PD) [25–28], including in post-mortem human samples. Acute neuronal injury from hypoxia-ischaemia [29] or traumatic insult [30] also promoted the formation of active necrosome (RIPK1/RIPK3/MLKL) and drove necroptotic neuronal death, and increased phosphorylation of MLKL is evident in the ischaemic core of human postmortem brain tissues [31]. In an integrated necroptosis-driven neurodegeneration hypothesis, necroptosis-susceptible neurons and oligodendrocytes would liberate danger-associated molecular patterns (DAMPs) or immunogenic factors to trigger microglial RIPK1-dependent neuroinflammatory transcription, thus generating a progressive inflammatory milieu to propagate CNS injury [25, 26].

#### Activation and targeting necroptosis in adult hypoxia-ischaemic brain injury

The very first evidence supporting that selective targeting of RIPK1 and necroptosis was therapeutically effective came from adult ischaemic brain injury study [29, 32]. In mice subjected to middle cerebral artery occlusion (MCAO), intracerebrovascular administration of RIPK1 inhibitor necrostatin-1 (Nec-1) reduced infarct size in a dose-dependent manner and even when given 6 h after the injury [29]. Serine 161 residue of RIPK1 was shown to be crucial for the inhibitory effect of Nec-1 on RIPK1 and necroptosis, and as an allosteric kinase inhibitor Nec-1 preferentially binds to the inactive conformation of RIPK1 to prevent RIPK1 activation [25, 32]. More recent study revealed that RIPK1 kinase mediates the sequential activation of necroptosis and apoptosis in the ischaemic penumbra in transient MCAO, with early phosphorylation of RIPK3 and MLKL in neurons and endothelial cells at 1–2 h of reperfusion, followed by caspase 3 cleavage in neurons at 5–24 h of reperfusion [33]. Moreover, RIPK1 kinase-dead mutation (homozygous RIPK1^D138N/D138N^) but not RIPK3 deficiency reduced ischaemic infarct and microgliosis in a murine model of transient MCAO [33]. In addition to RIPK1-targeted strategies, therapeutic agents with different mechanisms of action, such as TNF receptor-associated factor 2 (TRAF2, which suppresses death receptor signalling) [34], TrkB agonist (analogous to brain-derived neurotrophic factor, BDNF) [31], ex-527 (small molecule inhibitor of sirtuin 1) [35] and salubrinal (small molecule inhibitor that decreases protein translation and ER stress) [36], have been shown to also block necroptosis signalling and confer neuroprotection in rodent models of stroke.

#### Activation and targeting necroptosis in neonatal hypoxic-ischaemic brain injury

The pathogenic contribution of necroptosis neonatal H/I brain has also been studied, albeit to a much lesser extent when compared to studies on the adult brain. When using rodents to study neonatal H/I brain injury in termed neonates, postnatal day 7 to day 10 (PND7-10) mice and rats are deemed most appropriate and suitable, in view of the compelling evidences from cross-species comparison studies showing that the neurodevelopmental trajectories and signatures in 36 to 40-week in human correlate with PND7-10 in rodents [37–39], which are born altricial like human with significant brain development taking place after birth. In PND7 mice, i.c.v administration of the chemically improved Nec-1 analogue Nec-1s (Nec-1 stable, or 7-Cl-O-Nec-1) after cerebral H/I decreased cerebral, thalamic and hippocampal infarcts [40], suppressed RIPK1/NFκB activation with attenuated cytokine level (TNF-α, IL-1β, IL-6) [40] and improved mitochondrial function [41] at day 1 and day3 post-injury. Similarly, in PND7 rats increased necroptosis was detected in the ipsilateral hippocampus after unilateral carotid artery ligation and hypoxia (an established rodent model of HIE) [42]. Moreover, in PND6 rats, RIPK1 inhibition with necrostatin-1 before and after H/I brain injury provided additional benefits to therapeutic hypothermia, seen as reduced TNF-a response, MLKL phosphorylation and brain volume loss on MRI [43]. A hyperglycaemia-driven shift from apoptosis to necroptosis has also been described in neonatal H/I brain injury, with the hypothesis that aggravated mitochondrial reactive oxygen species (ROS) response from hyperglycaemia leads to RIPK1 oxidisation and oligomerisation, and subsequently promotes necrosome formation and necroptosis activation upon TNF-a engagement [44].

### Molecular regulation of necroptosis

**Fig. 2:**
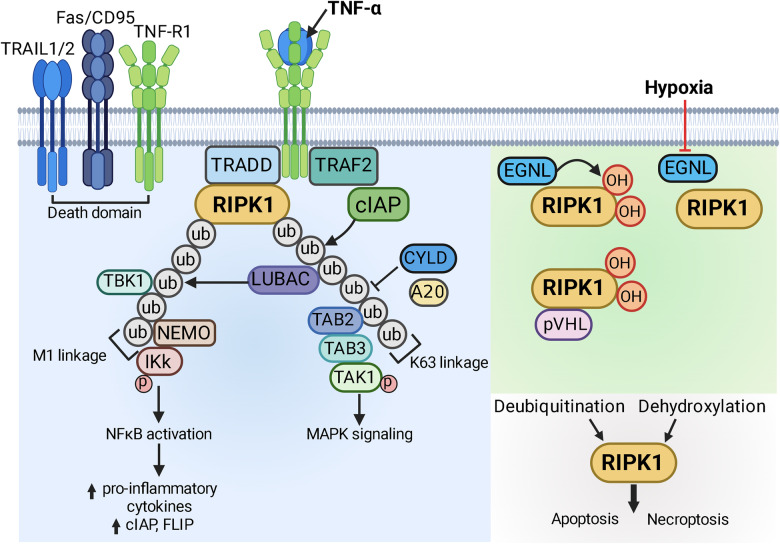
Post-translational modification of RIPK1. Binding of the tumour necrosis factor superfamily (TNFSF) to the tumour necrosis factor receptor superfamily (TNFRSF) leads to TRADD (TNFR1-associated DD protein)-dependent recruitment of receptor-interacting protein kinase (RIPK1) through homotypic interaction. The E3 ubiquitin ligase cellular inhibitor of apoptosis 1 or 2 (cIAP1/2) is subsequently recruited by TNFR-associated factor 2 (TRAF2) to promote K63 ubiquitination (ub, grey circles) of RIPK1 to form the K63 polyubiquitin linkage. The K63 linkage facilitates the recruitment and activation of transforming growth factor β-activated kinase 1 (TAK1) via TAK-associated binding protein 2 and 3 (TAB2/3) to induce the pro-survival MAPK signalling pathway. The presence of K63 linkage further promotes the recruitment of linear ubiquitination assembly complex (LUBAC) to lead to M1 (linear) polyubiquitination of RIPK1 (ub, grey circlesO. The M1 ubiquitin linkage is recognised by NFκB essential modulator (NEMO) and inhibitor of κB kinases (IκK) to stimulate the NFκB signalling pathway and upregulate the transcription of pro-inflammatory cytokines; transcription of anti-apoptotic proteins such as cIAP and c-FLIP (cellular FLICE (FADD-like IL-1β-converting enzyme)-inhibitory peptide) is also activated by NFκB. Endogenous deubiquitinases such as CYLD and A20 can also be recruited to dissemble the M1 and K63 polyubiquitin linkages. Moreover, TAK1 and TANK-binding kinase 1 (TBK1) could mediate the inhibitory phosphorylation of RIPK1. The aggregation and stabilisation of RIPK1 with the collection of post-translational editing enzymes is termed as *complex I*. In addition to poly-ubiquitination, proline residues on RIPK1 are hydroxylated (OH, red circles) by the prolyl hydroxylase enzyme Egl nine homologues (EGLNs) and the hydroxylated RIPK1 interacts with von Hippel Lindau protein (pVHL) to prevent downstream RIPK1 signalling. Hypoxia prevents EGLNs-mediated RIPK1 prolyl hydroxylation to avoid the negative regulation by pVHL. Taken together, when RIPK1 becomes deubiquitinated (e.g. cIAP inhibition by SMAC/DIABLO or SMAC synthetics, NEMO knockout or TAK1 knockout, upregulation of CYLD and A20) and dehydroxylated during hypoxia, RIPK1 is released from an “inactive” state and resumes its kinase activity to activate apoptosis or necroptosis pathways. cIAP1/2 cellular inhibitor of apoptosis 1 or 2, c-FLIP cellular FLICE inhibitory peptide, CYLD cylindromatosis tumour suppressor protein, DIABLO direct inhibitor of apoptosis-binding protein with low pI, EGLN Egl nine homologues, FLICE FADD-like IL-1β-converting enzyme, IκK inhibitor of κB kinases, LUBAC linear ubiquitination assembly complex, MAPK mitogen activated protein kinase, NEMO NFκB essential modulator, NFκB nuclear factor kappa B, OH hydroxylation, RIPK1 receptor interacting protein kinase 1, SMAC second mitochondria-derived activator of caspase, TAB2/3 TAK-associated binding protein 2 and 3, TAK1 transforming growth factor β-activated kinase 1, TBK1 TANK-binding kinase 1, TNFSF tumour necrosis factor superfamily, TNFRSF tumour necrosis factor receptor superfamily, TRADD TNFR1-associated DD protein, TRAF2 TNFR-associated factor 2, ub ubiquitination, pVHL von Hippel Lindau protein. Created with BioRender.com.

Necroptosis signalling begins with RIPK1 activation. Binding of tumour-necrosis factor superfamily ligands (TNFSF, e.g. tumour necrosis factor alpha – TNFα, Fas ligand - FasL) to the tumour-necrosis factor receptor superfamily (TNFRSF, e.g. TNF receptor, Fas) stimulates the death domain (DD) of receptor, leading to the recruitment DD-containing TNFR1-associated DD protein (TRADD) and RIPK1 via homotypic binding to form a membrane-bound *complex I* [45, 46] (Fig. 2). TRADD further recruits the E3 ubiquitin ligases cellular inhibitor of apoptosis 1 or 2 (cIAP1 or cIAP2) to promote K63 ubiquitination of RIPK1. The K63 ubiquitin chain on RIPK1 subsequently induces pro-survival MAPK signalling pathway and IκB (inhibitor of κB) kinases (IκK) to promote NFκB-dependent gene transcription, including the anti-apoptotic proteins cIAP1/2 and c-FLIP (cellular FLICE (FADD-like IL-1β-converting enzyme)-inhibitory peptide) and the pro-inflammatory cytokines, to simultaneously suppress cell death and propagate inflammation. Removal of ubiquitin chains by de-ubiquitinases CYLD and A20, together with cIAP1/2 degradation via autoubiquitination or by SMAC mimetic (synthetic compound that mimic the action of endogenous SMAC/DIABLO) [47], would release RIPK1 from complex I marking the irreversible commitment of RIPK1 to regulated cell death pathway.

In addition to ubiquitination/de-ubiquitination, prolyl hydroxylation was recently identified as an alternative posttranslational modification that could regulate RIPK1 activity [48]. Specifically, the prolyl hydroxylase enzyme Egl nine homologues (EGLNs) was shown to promote prolyl hydroxylation of RIPK1 at multiple proline residues to favour binding with pVHL and suppress RIPK1 activity under normoxia. Prolonged hypoxia alleviates RIPK1 suppression and triggers necroptosis by preventing EGLN-mediated prolyl hydroxylation, which is independent of the canonical death receptor pathway and represents a more direct and relevant mechanism by which RIPK1 activates necroptosis signalling during hypoxia-ishcaemic brain injury.

**Fig. 3:**
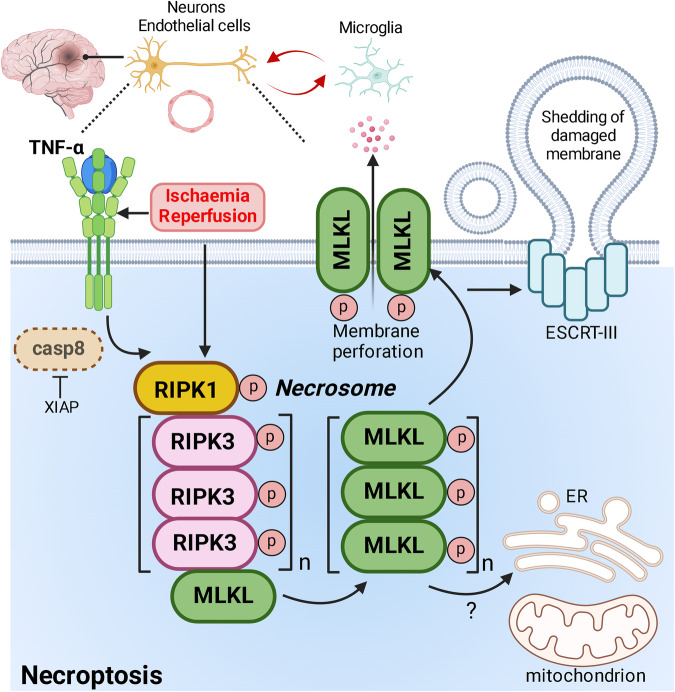
Activation of RIPK1-dependent necroptosis in ischaemia-reperfusion brain injury. In the hypoxic-ischaemic brain, neurons and endothelial cells are particularly susceptible to programmed cell death including necroptosis. Tumour necrosis factor-alpha (TNF-α) signalling through cell death receptor or direct activation of RIPK1 by cerebral ischaemia-reperfusion (I/R) initiates necroptosis, provided that RIPK1 is de-ubiquitinated and dehydroxylated (as shown in Fig. 2). When caspase-8 is absent or inhibited, for example, by endogenous X-linked inhibitor of apoptosis protein (XIAP), RIPK1 proceeds to recruit RIPK3 to facilitate RIPK3 oligomerisation (indicated by n) and trans-phosphorylation takes place between RIPK1 and RIPK3. Stabilised and phosphor-activated RIPK3 oligomer is necessary for the subsequent recruitment and activation of the pseudokinase mixed lineage kinase-domain like (MLKL), collectively forming the necrosome. RIPK3-mediated phosphorylation of MLKL leads to conformational change that is conducive for MLKL oligomerisation, whereby the MLKL oligomers can be comprised of three, four or six monomers (indicated by n). Oligomerised MLKL translocates to plasma membrane to perforate the lipid bilayer, the putative mechanism by which MLKL execute necroptosis. Necroptotic cell death is likely associated with release of immunogenic, cytosolic contents that could further stimulates the resident microglia to mount a pro-inflammatory response to propagate the neuro-inflammatory milieu (red double-way arrows). Moreover, membrane-localised MLKL may also interact with the endosomal sorting complex required for transport III (ESCRT-III) complex, to promote shedding of the damaged plasma membrane to preserve membrane integrity and delay necroptotic death. MLKL-containing necrosome may also translocate to the lipid membrane of endoplasmic reticulum and mitochondrion, the implication of which is currently being investigated. casp 8 caspase 8, ER endoplasmic reticulum, ESCRT-III endosomal sorting complex required for transport III, RIPK1 receptor-interacting protein kinase 1, RIPK3 receptor-interacting protein kinase 3, TNF-α tumour necrosis factor-alpha, XIAP X-linked inhibitor of apoptosis protein. Created with BioRender.com.

Upon its dissociation from complex I, RIPK1 could interact with FADD and pro-caspase 8 to form the death-inducing signalling complex [17–19, 47] (DISC, or *complex IIa*) to activate the pro-caspase 8 homodimers. When caspase 8 is inactivated (e.g. synthetic or viral caspase inhibitor) or absent, RIPK1 recruits RIPK3 [49] through their RIP-homotypic interaction motif (RHIM) to form *necrosome* [50] (Fig.3). RIPK1 and RIPK3 undergo autophosphorylation and trans-phosphorylation [51, 52], whereby phosphorylation of RIPK1 at Ser161 residue was shown to confer its kinase activity, and phosphorylation of Ser199 (Ser204 in mouse) and Ser227 (Ser232 in mouse) residues of RIPK3 is crucial for its pro-necroptotic activity [53, 54].

Subsequently, the kinase domain of RIPK3 associates with the C-terminal kinase-like domain of MLKL, before phosphorylating MLKL. Phosphorylation of MLKL at the specific C-terminal residues (T357 and S358 in human cells, and S345 in murine cells) [54, 55] alleviates the suppression of C-terminal pseudokinase domain on the N-terminal four α-helix bundle (4HBD) domain. After that, MLKL oligomerizes into high-molecular-weight oligomers before translocation to the plasma membrane [56]. The 4HBD domain of MLKL contains positive charged residues to interact with the negatively charged phospholipids of the plasma membrane (specifically, phosphoatidylinositol phosphates, PIP) to form pores and cause membrane permeabilisation [57, 58], the putative mechanism by which necroptosis executes lytic cell death.

While mechanistic studies into necroptosis signalling and related pathways have provided significant molecular insights (Fig. 3), RIPK1 remains the predominant if not sole target for necroptosis inhibition in hypoxic-ischaemic brain injury. On the other hand, RIPK3- or MLKL-targeted strategies have also been reported to be beneficial in acute and chronic injuries of the kidneys [59], liver [60], pancreas [61] and heart [62]. Overall RIPK3 inhibition/silencing conferred superior protection than MLKL deficiency, which has been attributed to RIPK3’s critical and necroptosis-independent role in apoptosis and inflammation, like RIPK1. Importantly, RIPK3 deficient mice and to a lesser extent MLKL knockout mice were also protected from blood-brain barrier damage and motor and cognitive functional decline after controlled cortical impact (CCI) injury [63]. In view of the above, RIPK3 inhibition represents a potential, alternative strategy for treating hypoxic-ischaemic brain injury to enable concomitant suppression of inflammation, apoptosis and necroptosis.

## Pyroptosis

Pyroptosis (pyro – fire or fever) describes an inflammatory type of regulated cell death that requires the proteolytic activation of pro-inflammatory caspase 1 and caspase 11 (human orthologs caspases 4 and 5) by the cytosolic inflammasome complexes that act as intracellular pathogen recognition receptors (PRRs) [19, 64, 65]. The most well-studied inflammasomes contain NLR-family pyrin containing domain 3 (NLRP3) or absent in melanoma 2 (AIM2) as the core scaffold protein, and are referred to as NLRP3 or AIM2 inflammasome [64]. Pyroptosis has been most extensively studied in macrophage and other phagocytic cell types of myeloid lineage (e.g. dendritic cell, microglia) and in the context of microbial infection and autoimmune condition[19, 65]. To a lesser extent, pyroptosis was also evident in epithelial cell, endothelial cell [66, 67] and neurons and was implicated in hypoxic-ischaemic and ischaemia-reperfusion injury of solid organs [67–70].

### Inflammasome and pyroptosis in adult hypoxia-ischaemic brain injury

Increased expression of NLRP3 inflammasome components, including NLR-family pyrin containing domain 3 (NLRP3), apoptosis-associated speck-like protein containing a caspase recruitment domain (ASC), interleukin-1 beta (IL-1β) and interleukin-18 (IL-18), were detected in the post-mortem samples from stroke patients [71]. In rodent model of stroke (i.e. transient MCAO), microglia and neurons were identified as the major sources of inflammasome activity to contribute to brain injury [72]. Specifically, upregulated expression of NLRP3, ASC, cleaved caspase-1, IL-1β and/or IL-18 were first detected in the microglia (6 h), only to precede increased inflammasome activity in neurons and endothelial cells at 24 h after transient ischaemia [67, 72]. Transient cerebral ischaemia also induced alternative signalling complexes including AIM2 and NLR family, CARD-domain containing 4 (NLRC4) inflammasomes to contribute to brain injury [73]. Genetic silencing of NLRP3 [72], NLRC4, AIM2 [73] or IL-1β converting enzyme (ICE) [74] was shown to reduce blood brain barrier leakage and/or ameliorate brain infarct volume. Studies also confirmed gasdermin D (GSDMD)-driven pyroptosis and subsequent inflammatory response as targetable mechanisms in hypoxic-ischaemic brain injury [75]. Global GSDMD knockout in mice reduced neuronal loss, brain infarct and pro-inflammatory cytokine IL-1β release and prevented peripheral immune infiltration following transient cerebral ischaemia. Post-stroke administration of disulfiram that inhibits GSDMD pore formation and pyroptosis-related IL-1β release also prevented brain lesion and preserved neurological functions [75]. Similarly for traumatic brain injury, nanoparticle-packaged disulfiram was shown to suppress pyroptotic cell death, reduce astrocytic and microglial activation, maintain BBB integrity and preserve motor and cognitive function [76].

### Inflammasome and pyroptosis in neonatal hypoxic-ischaemic brain injury

**Fig. 4:**
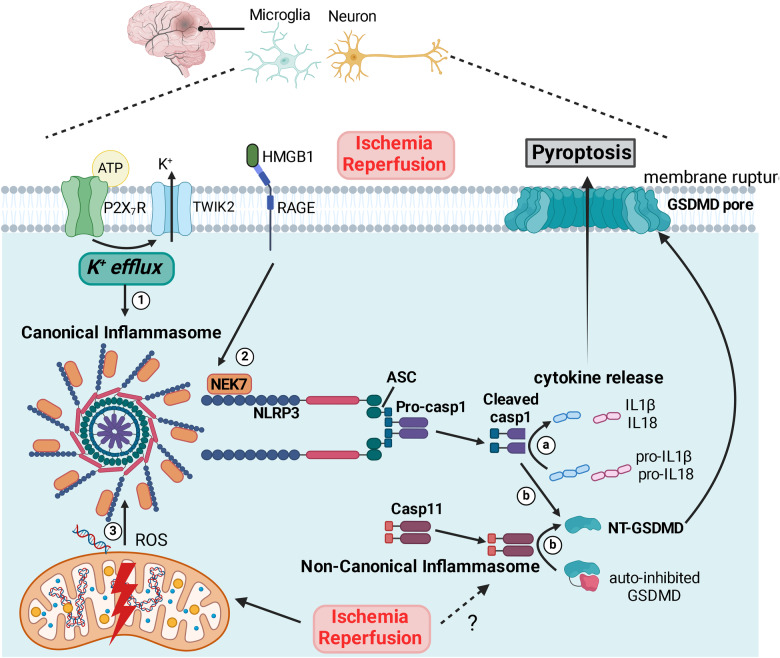
Activation of inflammasome and pyroptosis in ischaemia-reperfusion brain injury. Following cerebral ischaemia and reperfusion, microglia and neurons have been shown to be prone to pyroptosis, and the concurrent release of mature interleukins make pyroptosis a highly pro-inflammatory type of programmed cell death. Hypoxia-ischaemia (H/I) challenge could trigger inflammasome assembly and pyroptosis through shared molecular triggers (see below) implicated in infectious and inflammatory conditions, and/or by activating of mechanism(s) specific for H/I and I/R that warrant future studies. Of the different canonical inflammasome configurations, NLRP3 inflammasome is the most well-characterised complex and comprises of NLR-family pyrin containing domain 3 (NLRP3), apoptosis-associated speck-like protein containing a caspase recruitment domain (ASC) and pro-caspase 1, the oligomerisation of which is facilitated by NIMA-related kinase 7 (NEK7). Assembly of the canonical inflammasome complex can be triggered by different stimuli converging at the level of K^+^ efflux (1). For example, extracellular ATP released during I/R could act as danger-associated molecular pattern (DAMP) to activate purinergic receptor P2X_7_ (P2X7R) and subsequently results in K^+^ efflux via tandem pore domain weak inward rectifying K^+^ channel (TWIK2). High-mobility group box 1 (HMGB1) was demonstrated as another DAMP that activates NLRP3 inflammasome during neonatal H/I brain injury, by signalling through receptor for advanced glycation end product (RAGE) (2). Moreover, cerebral I/R-induced mitochondrial stress and damage could lead to accumulation of ROS or mitochondrial DNA release (3), which had also been shown to drive NLRP3 inflammasome assembly. Subsequently, cleaved caspase-1 processes pro-interleukin 1beta (pro-IL1β) and pro-interleukin 18 (pro-IL18) to generate mature cytokines for extracellular release (a). Activated caspase-1 additionally cleaves gasdermin D (GSDMD) to promote N-terminal (NT)-GSDMD oligomersiation and pore formation on the plasma membrane (b), leading to pyroptosis and concurrent cytokine release. It remains unclear if the non-canonical inflammasome comprising of caspase-11 is activated during cerebral I/R and contributes to I/R-induced pyroptosis (b). ASC apoptosis-associated speck-like protein containing a caspase recruitment domain, GSDMD gasdermin D, H/I hypoxia-ischaemia, HMGB1 high mobility group box 1, IL-1β interleukin-1 beta, IL-18 interleukin-18, I/R ischaemia-reperfusion, LPS lipopolysaccharides, NEK7 NIMA-related kinase 7, NLRP3 NLR-family pyrin containing domain 3, ROS reactive oxygen species, P2X_7_R purinoceptor P2X_7_, RAGE receptor for advanced glycation end product, TWIK2 tandem pore domain weak inward rectifying K^+^ channel. Created with BioRender.com.

NLRP3 inflammasome activity and pyroptosis have also been implicated in ischaemia-reperfusion injury of the neonatal brain. In HIE neonates, peripheral blood levels of NLRP3, caspase-1, IL-1β and GSDMD were elevated compared to controls and the increments correlated with clinical severity of HIE (lower Apgar scores) [77]. In rodent model of HIE, expressions of NLRP3, ASC, cleaved caspase-1, mature IL-1β and GSDMD were elevated in the ischaemic hemisphere [77, 78]. Both the microglial and neuronal populations were shown to be susceptible to pyroptosis together with a pro-inflammatory shift of the microglial population [77]. So far, it is unclear whether cerebral H/I triggers the same set of molecular mechanisms (e.g. K^+^ efflux, mitochondrial stress, see later in molecular regulation; Fig. 4) implicated in infectious and inflammatory conditions to induce inflammasome and pyroptosis or that H/I-specific mechanisms also exist. One study demonstrated the DAMP molecule high mobility group box 1 (HMGB1) as a potent inducer of pyroptosis in microglia in neonatal rats after cerebral hypoxia-ischaemia, whereby pharmacological inhibition of the HMGB1/ receptor for advanced glycation end products (RAGE)/cathepsin B signalling pathway attenuated pyroptosis and hippocampal injury and restored spatial memory [79].

### Molecular regulation of pyroptosis

Inflammasome-mediated inflammatory caspase maturation and cytokine release likely evolved as a unique innate defense mechanism against microbial infections. Innate immune system relies on both transmembrane and intracellular pathogen recognition receptors (PRRs) to sense pathogen-associated molecular patterns from pathogens (microbial nucleic acids, bacterial cell wall) and danger-associated molecular patterns (DAMPs) from damaged host cells (heat shock protein 70/90 – HSP70/90, ATP, high-mobility the group box 1 – HMGB1, uric acids), in order to mount a pro-inflammatory response to clear infection and remove damaged cells [64, 65, 80]. Cytosolic PRRs including nucleotide-binding oligomerisation domain and leucine-rich repeat-containing proteins (NLRs), absent in melanoma 2 (AIM2)-like receptor (ALR) and retinoic acid-inducible gene (RIG)-I-like receptor (RLR) are activated by intracellular signals [64].

The “canonical” inflammasome functions as a capase-1 activation platform [64, 80, 81]. The sensor NLRP3 oligomerises and recruits the adaptor protein apoptosis-associated speck-like protein containing a caspase recruitment domain (ASC) to engage pro-caspase 1 in the presence of NEK7, to facilitate the auto-cleavage of pro-caspase-1 into catalytically active caspase-1 [19, 64, 65, 80]. Cleaved caspase-1 assumes complete cysteine protease activity to process the pro-inflammatory cytokines interleukin-1 beta (IL-1β) and interleukin-18 (IL-18) for extracellular release [19, 64, 65]. A large number of unrelated molecular triggers has been identified as triggers for NLRP3 inflammasome, including endogenous DAMPs (e.g. ATP, mtDNA), environmental particulates (e.g. alum, uric acid crystals), microbial toxin/nucleic acid [80], phagocytosed particulates [82, 83], mitochondrial ROS [84, 85] and mitochondrial DNA [86] released into the cytosol.

Studies also identified a “non-canonical” inflammasome that specialises in the activation of caspase-11 (human caspase-4/-5) upon lipopolysaccharides (LPS) stimulation. Unlike caspase-1, activated caspase-11 only weakly cleaved IL-18 without activity towards IL-1β [87, 88]. The discovery of gasdermin-D (GSDMD) as the shared cleavage substrate of caspase-1 [89] and caspase-11 [90] united the canonical and non-canonical inflammasome pathways towards the execution of pyroptosis. Caspase-mediated cleavage of the C-terminus relieves GSDMD from an auto-inhibited state to enable its translocation to the plasma membrane [91]. Upon membrane localisation, GSDMD oligomerises directly and binds to phosphatidylinositol and phosphatidylserine lipids to form nano-sized pores to cause cell lysis [92], a mechanism that is reminiscent of MLKL-mediated necroptosis.

## Ferroptosis

Ferroptosis specifically describes a form of regulated necrosis featuring iron-dependent lethal lipid peroxidation [93, 94]. Since its initial discovery in the development of anti-cancer therapy [95, 96], ferroptosis has been shown to contribute to a variety of human diseases including hypoxic-ischaemic injury (in kidneys, liver, and brain) and neurodegenerative conditions [19, 93].

### Activation and targeting ferroptosis in neonatal hypoxic-ischaemic brain injury

**Fig. 5:**
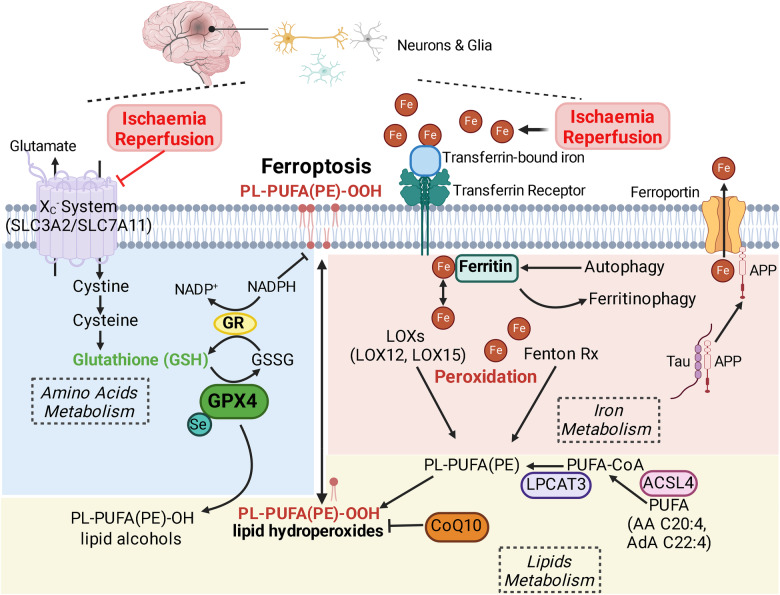
Activation of ferroptosis in ischaemia-reperfusion brain injury. Compared to the adult brain, neonatal brain maybe more susceptible to hypoxia/ischaemia-induced ferroptosis owing to a higher poly-unsaturated fatty acids (PUFA) content prone to lipid peroxidation (lipids metabolism) and/or reduced antioxidation capacity from lower level of glutathione (amino acids metabolism). Common to both the adult and developing brains, increased brain iron deposition (iron metabolism) from ischaemia-reperfusion (I/R) will sensitise different cell types to ferroptosis. In the hypoxic-ischaemic brain, neuron and glia populations have been shown to be prone to ferroptosis. Ferroptosis is tightly regulated by balancing the metabolisms of amino acids, lipids and free iron. Glutathione (GSH) is required by the antioxidative enzyme glutathione peroxidase 4 (GPX4) to facilitate the conversion of toxic phospholipid hydroperoxides into non-toxic lipid alcohols therefore antagonising ferroptosis. The rate-limiting step in GSH biosynthesis is the extracellular import of cystine through Xc^−^ system (SLC3A2/SLC7A11). Excitotoxicity and high extracellular concentration of glutamate during cerebral I/R may hinder glutamate/cystine exchange to impair the biosynthesis of GSH. This in turn results in accumulation of phospholipid hydroperoxides at plasma membrane to cause membrane damage and drive ferroptosis. Endogenous antioxidants such as coenzyme Q10 (CoQ10) could also attenuate lipid peroxidation and ferroptosis. Cerebral I/R also leads to increased iron deposition in the brain, in part due to breaching of the blood–brain barrier, to sensitise susceptible cells to ferroptosis. Free iron ions are transported via transferrin receptor and intracellular iron concentration is regulated through binding to ferritin. Moreover, selective degradation of ferritin during autophagy (e.g. ferritinopathy) increases the cellular pool of labile iron ions to promote ferroptosis. In the brain, tau-mediated amyloid precursor protein (APP) transport to the plasma membrane to was shown to stabilise ferroportin (Fpt) and iron efflux. Labile iron ions enhance oxidation and peroxidation of poly-unsaturated fatty acids (PUFA) through Fenton reaction or lipooxygenases (LOX, e.g. LOX12, LOX15). Cellular pool of peroxidation-prone PUFAs, such phospholipid PUFA species containing the phosphotidylethanolamine moiety (PL-PUFA-(PE)) determines the cellular sensitivity to ferroptosis, and the readily oxidisable phospholipids are synthesised from PUFA in a two-step reaction involving acyl-coA synthetase long-chain family 4 (ACSL4) and lysophosphatidylcholine acyltransferase 3 (LPCAT3). ACSL4 acyl-coA synthetase long-chain family 4, APP amyloid precursor protein, CoQ10 coenzyme Q10, Fpt ferroportin, GPX4 glutathione peroxidase 4, GR glutathione reductase, GSH glutathione, H/I hypoxic-ischaemic, LOXs lipooxygenases, LPCAT3 lysophosphatidylcholine acyltransferase 3, NADP^+^ oxidised nicotinamide adenine dinucleotide phosphate, NADPH reduced nicotinamide adenine dinucleotide phosphate, PUFA poly-unsaturated fatty acids, PL-PUFA-PE phospholipid PUFA with phosphotidylethanolamine, PL-PUFA-PE-OH phospholipid PUFA alcohols with phosphotidylethanolamine, PL-PUFA-PE-OOH phospholipid PUFA hydroperoxides with phosphotidylethanolamine. Created with BioRender.com.

Compared to the adult brain, the immature brain may be more susceptible to ferroptosis in view of its high poly-unsaturated fatty acid (PUFA) content and limited antioxidant capacity [97, 98]. In both the adult and developing brains, H/I-induced excitotoxicity and high extracellular concentration of glutamate may inhibit glutamate/cystine exchange to impair the biosynthesis of antioxidant glutathione (GSH)(See below in molecular regulation). Moreover, blood–brain barrier compromise from H/I challenge would facilitate peripheral iron deposition in the brain. (Fig. 5).

In severely asphyxiated newborns, plasma levels of non-protein bound free iron and lipid oxidation were increased in the first 24 h of birth, and elevation of plasma free iron correlated with neurodevelopmental outcome at 1 year of age [99]. Moreover, cerebrospinal fluid (CSF) from HIE infants also contained higher level of free iron and lipid oxidation end-product malondialdehyde (MDA) [100]. In a rat model of HIE, early iron deposition was evident in the ischaemic brain with increased iron staining detected in neurons and active glia that persisted for 3 weeks [101]. In the injured cerebral cortex, transferrin receptor and ferritin expressions were upregulation along with an increased level of MDA [102, 103], whereas expression of the ferroptosis inhibitor glutathione peroxidase 4 (GPX4) was downregulated together with decreased level of its antioxidant product GSH [102]. Administration of the iron chelator deferoxamine (DFO) or ferroptosis inhibitor ferrostatin-1 (Fer-1) to neonatal rats before H/I or upon reperfusion attenuated ipsilateral brain lesion and attenuated learning and memory deficits, whereas iron supplementation aggravated brain injury following HIE [102, 104]. Moreover, cerebral H/I associated GPX4 suppression could be reversed by resveratrol treatment, which signals through the anti-oxidant transcriptional factor nuclear factor erythroid-2-related factor 2 (Nrf2) to restore GPX4 expression and confer neuroprotection [105]. As a complementary strategy to iron lowering/chelation, preventing membrane lipid peroxidation through supplementation of 7-dehydrocholesteroal (7-DHC), a highly oxidisable lipid that spares phospholipids from peroxidation, also attenuated cell death, brain infarct and MDA level in neonatal rats after cerebral H/I [106].

### Activation and targeting ferroptosis in adult hypoxic-ischaemic brain injury

Ferroptosis and iron imbalance have also been implicated in I/R injury of the adult brain, whereby compromised BBB and upregulated transferrin could promote iron deposition iron from peripheral blood [107], and iron chelator (e.g. DFO) was demonstrated to be neuroprotective [107, 108]. Magnetic resonance imaging (MRI) study in ischaemic stroke patients confirmed abnormal iron deposition in different brain regions [109]. Studies on the adult brain yielded important mechanistic insights into how I/R brain injury impairs iron homoeostasis to drive ferroptosis. Tau protein was shown to promote brain iron efflux by trafficking the amyloid precursor protein (APP) to stabilise membrane iron exporter ferroportin (Fpn) [110]. Transient middle cerebral artery occlusion (MCAO) in 3-month-old mice acutely decreased brain Tau level to precede iron accumulation in the ipsilateral hemisphere [111], such that suppression of Tau-iron export axis could sensitise I/R-injured brain to ferroptotic cell death. Unexpectedly, hemispheric iron deposition and brain volume loss from MCAO were attenuated in Tau-KO mice, to suggest upregulation of a compensatory iron export mechanism to confer resistance to Tau suppression-associated iron toxicity following MCAO. On the other hand, Tau-deficient 12-month-old mice displayed age-dependent iron accumulation that could be due to long-term disruption to brain iron homoeostasis, and transient MCAO did not further enhance iron deposition in the injured hemisphere [111]. Notwithstanding, ferroptosis-targeted interventions reduced brain infarct and improved functional outcomes in both the juvenile and aged mice and in the Tau-deficient background, by promoting iron efflux with ceruloplasmin (Cp) or soluble APP [111]. Moreover, the mitochondrial iron storage protein mitochondrial ferritin (MtFt) was shown to be neuroprotective during transient MCAO [112], such that MtFt silencing exacerbated MCAO-associated iron accumulation, lipid peroxidation and brain infarct, whereas MtFt overexpression reduced iron deposition and improved functional recovery [112].

### Molecular regulation of ferroptosis

Ferroptosis was firstly described in cancer cells treated with the RAS-selective lethal small molecule erastin [95], which inhibits the glutamate/cystine antiporter (system Xc^-^) to prevent cellular cystine intake. System Xc^-^ is a cell surface transporter comprised of solute carrier family 3 member 2 (SLC3A2) and solute carrier family 7 member 11 (SLC7A11) and exchanges glutamate for extracellular cystine at a 1:1 ratio, and intracellular cystine is reduced to cysteine to feed into glutathione (GSH) biosynthesis [113]. As the rate-limiting step in GSH synthesis, depriving cells of cystine by system Xc^−^ inhibitor erastin depletes the cellular reservoir of GSH and sensitises cells to iron-dependent lipid peroxidation and oxidised cell death [95]. GSH is utilised by the enzyme glutathione peroxidase 4 (GPX4) as a reducing agent to convert the toxic phospholipid hydroperoxides (PL-OOH) into harmless alcohol derivatives (PL-OH) [94, 114]. Inactivation of GPX4 by specific small molecule inhibitor (1S, 3R)-RSL3 (RSL3) or genetic silencing of GPX4 provoked excessive lipid peroxidation and cell death that are reminiscent of cystine depletion by erastin [94, 114, 115].

The requirement for free iron ions to induce lipid peroxidation and ferroptosis was confirmed by the observation that addition of iron chelators (e.g. deferoxamine, DFO; ciclopirox, CPX) prevented cell death [96]. Once inside the cells, free iron is modulated by the carrier protein ferritin, which introduces a potential checkpoint for autophagy to regulate ferroptosis. Autophagy-mediated selective degradation of ferritin is achieved through the specific cargo receptor nuclear receptor coactivator 4 (NCOA4) that delivers ferritin to autophagosome [116, 117], thus freeing irons to promote lipid peroxidation and cell death in a process termed as ferritinophagy.

Labile iron accelerates lipid peroxidation via enzyme-independent Fenton reaction or non-heme, iron-containing lipooxygenases (LOXs). During Fenton reaction, ferrous ions (Fe^2+^) react with hydrogen peroxides (H_2_O_2_) to generate hydroxyl free radicals (OH•) that subsequently oxidise lipids [98, 118]. Lipoxygenases are iron-containing enzymes that catalyse the oxidation/peroxidation of poly-unsaturated fatty acids (PUFAs) [93, 94], and arachidonate-12 lipoxygenase (12-LOX) [119] and arachidonate-15 lipoxygenase (15-LOX) [120] have been shown to be crucial to peroxidation of free PUFA and PUFA-phospholipids during ferroptosis. The pool of PUFA phospholipids are synthesised from free PUFAs involving a two-step reaction: acyl-coA synthetase long-chain family 4 (ACSL4)-dependent conversion of free PUFA (e.g. AA, AdA) into PUFA-CoA acyl [121], which is then incorporated into the phospholipid backbone by lysophosphatidylcholine acyltransferase 3 (LPCAT3). Taken together, the sensitivity towards ferroptosis is dictated by the cellular pool of readily oxidisable PUFA phospholipids, for example, genetic knockdown of ACSL4 [121] or LPCAT3 [122] conferred resistance against ferroptosis whereas PUFA supplementation sensitised cells to ferroptosis [122].

## Autophagy and mitophagy

Autophagy refers to a tightly regulated cellular catabolic process that enables continuous renewal of cellular components under basal condition as well as turnover of damaged or undesirable cellular fractions during stress [123, 124]. An important distinction should be made between non-selective macroautophagy and selective autophagy. The former is considered to be a predominantly starvation response that remodels cellular contents and redistributes energy to adapt to a low-nutrition environment, whereas the latter permits targeted removal of dysfunctional organelles and protein aggregates to avoid further cellular injury [125]. Since its discovery, studies have regarded the upregulation of autophagy as a likely cytoprotective mechanism during stress and starvation, along with the identification of different selective autophagy pathways that turnover specific organelles (e.g. endoplasmic reticulum – reticulophagy, peroxisome – pexophagy, and mitochondrion – mitophagy) [125, 126].

Mitophagy is typically preceded by mitochondrial fission to divide mitochondria into suitable sizes or to expel damaged mitochondrial fractions from the mitochondrial pool for engulfment by autophagosome [127, 128]. In mammalian cells, mitophagy (selective autophagy of mitochondria) is mediated by specific mitophagy receptors (e.g. Bcl-2 interacting protein 3, BNIP3 and Bcl-2 interacting protein 3-like, BNIP3L/NIX) or through PTEN-induced putative kinase 1 (PINK1)/Parkin-mediated ubiquitination pathway (details see below). Both pathways are capable of engaging with the ATG8-family proteins (LC3, GABARAP) through conserved LC3-interacting region (LIR) motif to recruit autophagosomal membranes encapsulate mitochondria [128].

### Autophagy and mitophagy in adult hypoxic-ischaemic brain injury

**Fig. 6:**
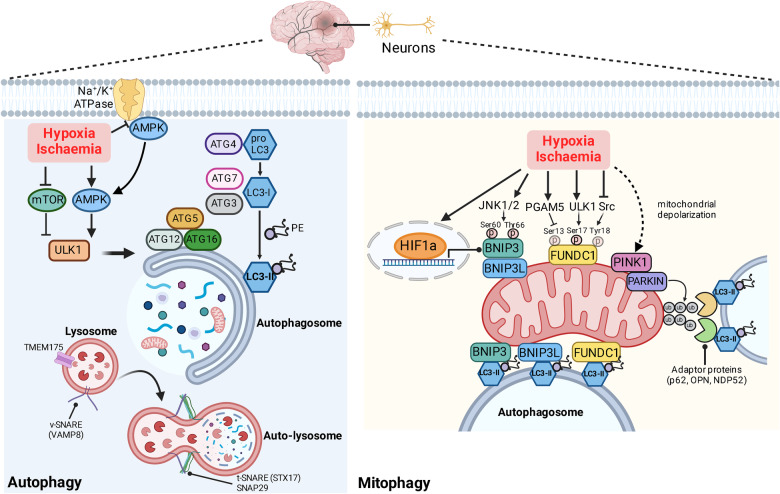
Activation of autophagy and mitophagy during ischaemia-reperfusion brain injury. Hypoxia-ischaemia (H/I) potently activates key autophagy and mitophagy pathways, and studies on H/I brain injury have mostly focused on neuronal autophagy and mitophagy. Based on animal models, an important distinction between H/I-only (i.e. permanent MCAO) and ischaemia-reperfusion (I/R; i.e. transient MCAO or HIE) brain injury warrants attention, whereby activation of autophagy and/or mitophagy is mostly beneficial in the I/R paradigm and could be exploited as a neuroprotective strategy. Hypoxia-ischaemia activates Atg1/Unc-51 like autophagy activating kinase 1 (ULK1) and initiate isolation membrane formation through mechanistic target of rapamycin (mTOR) and AMP-activated protein kinase (AMPK) dual regulation. The plasma membrane sodium potassium ATPase (Na^+^/K^+^ ATPase) was identified as a novel regulator of AMPK in I/R brain injury, whereby hypoxia disrupts the interaction between Na^+^/K^+^ ATPase and AMPK to reinstate the kinase activity of AMPK and trigger autophagy. Cerebral ischaemia-reperfusion challenge also upregulates the core autophagy machineries, including microtubule-associated protein 1 light chain 3-II (LC3-II, lipidated with phosphatidylethanolamine, PE) and p62/sequestosome and the lysosome marker lysosomal membrane protein 2 (LAMP2). In addition, the lysosomal ion channel transmembrane protein 175 (TMEM175) is critical to (auto)lysosomal function and protects against neonatal I/R brain injury. In receptor-mediated mitophagy, hypoxia-ischaemia directly promotes the transcription and expression of BNIP3 and BNIP3L through hypoxia-inducible factor alpha (HIF-1α), to result in their increased mitochondrial localisation and BNIP3/BNIP3L-dependent mitophagy. Hypoxia also induces JNK1/2 signalling to phosphorylate BNIP3 at serine 60/threonine 66 residues to stabilise BNIP3 and promote mitophagy. Fun14-domain containing 1 (FUNDC1) is another hypoxia-sensitive mitophagy receptor and its phosphorylation status is finetuned by PGAM5, ULK1 and Src kinase, whereby dephosphorylation of serine 13 and tyrosine 18 and phosphorylation of serine 17 are necessary for driving FUNDC1-dependent mitophagy during hypoxia. Moreover, hypoxia/ischaemia could induce mitochondrial depolarisation that could indirectly trigger PINK1/Parkin-mediated ubiquitin-dependent mitophagy pathway, although direct evidence on hypoxia-induced PINK1/Parkin pathway is currently lacking in the context of H/I brain injury (dashed line). The intrinsic mitophagy receptors recruits LC3-II autophagosome to mitochondria destined for degradation via their LC3-interacting region (LIR), and similarly poly-ubiquitinated mitochondrial substrates could engage with LC3-II autophagosome via adaptor proteins (e.g. p62). AMPK AMP-activated protein kinase, BNIP3 B cell lymphoma 2 Interacting Protein 3, BNIP3L/NIX B cell lymphoma 2 Interacting Protein 3-like, FUNDC1 Fun14-domain containing 1, H/I hypoxia-ischaemia, HIE hypoxic-ischaemic encephalopathy, HIF-1α hypoxia inducible factor-1 alpha, I/R ischaemia-reperfusion, JNK1/2 c-Jun N-terminal kinase 1/2, LAMP2 lysosomal membrane protein 2, LC3 microtubule-associated protein 1 light chain 3, LIR LC3-interacting region, MCAO middle cerebral artery occlusion, mTOR mechanistic target of rapamycin, NDP52 nuclear dot protein 52, OPTN optineurin, TMEM175 transmembrane protein 175, PE phosphatidylethanolamine, PGAM5 phosphoglycerate mutase/protein phosphatase 5, PINK1 phosphatase and tensin/PTEN homologue-indced kinase 1, ULK1 Atg1/Unc-51 like autophagy activating kinase 1. Created with BioRender.com.

Studies have reported increased mitophagy and upregulated expression of autophagy machineries in in vitro and in vivo models of hypoxic-ischaemic brain injury (i.e. stroke), with the most prominent increases seen during the reperfusion phase [129, 130] (Fig. 6). Importantly, inhibition of autophagy (3-methyladenine, bafilomycin A_1_, Atg7 or Atg5 knockdown) or mitophagy (mdivi-1, knockdown of PARK2 or BNIP3L) during reperfusion aggravated neuronal death after oxygen-glucose deprivation (OGD) and enlarged infarct volume in mice subjected to transient middle cerebral artery occlusion (tMCAO) [129]. On the other hand, blocking autophagy/mitophagy during OGD and in permanent MCAO (pMCAO) did not exacerbate neuronal injury and tissue loss [129]. These findings support autophagy and mitophagy as neuroprotective mechanisms activated by cerebral reperfusion. Parkin (PARK2) and Bcl-2 interacting protein 3-like (BNIP3L)/NIX-mediated mitophagy pathways are independently activated in cerebral ischaemia-reperfusion injury, and mice deficient in both pathways displayed the most extensive infarct compared to PARK2 or BNIP3L single-KO cohorts; forced expression of PARK2 effectively rescued neuronal viability and reduced infarct size in BNIP3L-KO subjects and vice versa [130]. Moreover, Parkin-dependent mitophagy could be potently induced by endoplasmic reticulum (ER) stress inducers tunicamycin and thapsigargin [131] or CO_2_ acidic post-conditioning, to lead to reduced infarct and neuronal death in mice following tMCAO [132].

The mechanisms by which hypoxia-ischaemia and subsequent reperfusion promote autophagy and mitophagy (BNIP3/BNIP3L and FUNDC1; See below in “Receptor-mediated mitophagy”) are complex and interconnected. Hypoxia/ischaemia, as with glucose and nutrient starvation, initiate the autophagy programme by activating the Atg1/Unc-51 like autophagy activating kinase 1 (ULK1) through mechanistic target of rapamycin (mTOR) and AMP-activated protein kinase (AMPK) dual regulation [133], thus enabling the formation of isolation membrane. More recently, the transmembrane protein pump Na+/K-ATPase (NKA) was demonstrated as a novel upstream regulator of autophagy during ischaemia-reperfusion brain injury, by signalling through the energy-sensing kinase 5′-adenosine monophosphate-activated protein kinase (AMPK) [134]. Hypoxia was shown to weaken the NKA/AMPK interaction at cell membrane to free AMPK and drive AMPK-induced autophagy, and NKA-binding antibody induced conformational change to NKA further disrupted such interaction to potentiate autophagy and afford neuroprotection, which were abolished by the autophagy inhibitor 3-MA.

### Autophagy and mitophagy in neonatal hypoxic-ischaemic brain injury

The increased plasma levels of the autophagy protein ATG5 and mitophagy mediator Parkin were detected in newborns diagnosed with HIE, and their concentrations were correlated with HIE severity, metabolic acidosis, requiring resuscitation at birth and a lower Apgar score [135]. In neonatal rodents, autophagy and mitophagy are strongly co-upregulated in the brain after cerebral hypoxia-ischaemia, with increased LC3-II conversion and co-localisation with the mitochondrial structural protein TOM20 and the lysosome marker lysosomal membrane protein 2 (LAMP2) [136–139]. Enhanced autophagy in cerebral cortex and hippocampus was concluded to be neuroprotective in the injured hemisphere, as autophagy inhibitor 3-MA deteriorated brain injury and biased cell death towards necrosis, whereas autophagy inducer rapamycin (which de-inhibits mTORC1-mediated blockade on autophagy initiation complex) reduced necrotic cell death and brain injury [136]. Additionally, enhancing auto-lysosomal degradation downstream to autophagy activation was also shown to be neuroprotective in neonatal H/I brain injury, whereby overexpression of the lysosomal transmembrane protein TMEM175 attenuated hippocampal neuron loss and preserved cognitive functions [140]. Contrasting reports on neonatal mice deficient in autophagy inducer ATG7 concluded protection from H/I brain injury with suppressed autophagosome formation and caspase-3 activation [138]; in this case, it would be difficult to determine whether the neuroprotection is attributed to inhibition of apoptosis, autophagy or both. Moreover, H/I-induced mitophagy likely occurs in two phases, with the first wave taking place within 24 h and is accompanied by upregulation of PINK1/Parkin, NIX and FUNDC1, while the second phase of mitophagy at 7 days post-H/I primarily features the PINK1/Parkin pathway [139].

### Molecular regulation of autophagy

All forms of autophagy require the formation of a double-membrane vesicular autophagosome, which derives from the progressive expansion of a small, flattened isolation membrane within the cytoplasm, before the subsequent engulfment of cellular components/organelles by the autophagosome. In mammalian cells, the enclosed autophagosome is transported to lysosomes and the inner autophagosomal membrane fuses with the lysosomal membrane, enabling the formation of autolysosome and the degradation of the sequestered materials by various hydrolases [123, 124, 127, 128, 141].

**Fig. 7:**
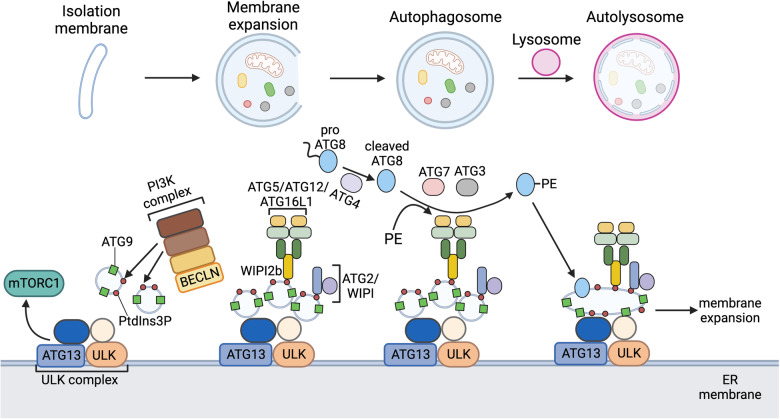
Biogenesis of autophagosome. In mammalian cells, autophagosome biogenesis commences at the endoplasmic reticulum subdomains with the formation of an isolation membrane. The isolation membrane continues to expand and bend into a spherical shape, while various intracellular components are encapsulated into the expanding membrane, before the isolation membrane completely closes to form the mature autophagosome. The outer autophagosomal membrane fuses with lysosome to deliver the sequestered contents for degradation in the acidic environment of autolysosome. Autophagosome biogenesis is intricately coordinated by numerous autophagy-related gene (ATG) proteins, and can be triggered during cellular starvation, hypoxia, and endoplasmic reticulum stress. The dissociation of mTORC1 from the ULK/ATG13 complex initiates isolation membrane formation at the endoplasmic reticulum (ER) subdomain. ATG9-containing vesicles are recruited to the ULK/ATG13 complex, and the PI3K complex generates phosphatidylinositol 3-phosphate (PtdIns3P) to be inserted into the ATG9-vesicles for recruitment of downstream ATGs. ATG2/WD-repeat protein interacting with phosphoinositides (WIPI) complex is recruited to the ATG9 vesicles via PtdIns3P to facilitate membrane expansion. The ATG12/ATG5/ATG16L1 complex can be localised to the initiation membrane to catalyse the lipidation of ATG8-family proteins (LC3A/B, GABARAP), through the linkage WIPI2b that recognises PtdIns3P. In this regard, conjugation with the lipid phosphatidylethanolamine (PE) is required for insertion of the ATG8-family proteins into the expanding isolation membrane. ATG8-family protein is first cleaved by ATG4, before the lipidation reaction is sequentially catalysed by ATG7, ATG3 and ATG12/ATG5/ATG16L1 complex. Once anchored to the precursor autophagosome membrane, ATG8-family protein could trigger membrane tethering and expansion, recruit autophagy receptors on autophagic cargoes (e.g. mitophagy), as well as promote autophagosome-lysosome fusion. ATG autophagy-related protein, ER endoplasmic reticulum, GABARAP GABA type A receptor-associated protein, LC3 microtubule-associated protein 1 light chain 3, mTORC1 mammalian target of rapamycin complex 1, PE phosphatidylethanolamine, PtdIns3P phosphatidylinositol 3-phosphate, ULK unc-51 like autophagy activating kinase, WIPI WD-repeat protein interacting with phosphoinositides. Created with BioRender.com.

Autophagosome biogenesis is an intricately regulated processes mediated by autophagy-related (ATG) genes and the ATG proteins they encode, which are highly conserved across eukaryotes and nearly 20 core ATGs have been identified to coordinate the highly intricate process of autophagosome synthesis [142] (Figs. 6 and 7), ultimately leading to the expansion/maturation of autophagosome membranes and lipidation of ATG8 for membranal insertion. In mammals, ATG8-family proteins consist of microtubule-associated protein light chain 3 (LC3) isoforms and GABA receptor-associated proteins (GABARAPs), which are conjugated with the lipid phosphatidylethanolamine (PE) for insertion into the autophagosome membrane. ATG8-family proteins are firstly cleaved by ATG4 to expose their C-terminals, before ATG3, ATG7 and the ATG12/ATG5/ATG16L1 complex acting sequentially as E1, E2 and E3 enzymes to conjugate PE to ATG8-family proteins. Once anchored to the autophagosome precursor, ATG8-family proteins then stimulate further membrane expansion and elongation, recruit autophagy receptors containing the LC3-interacting region (LIR), and facilitate the fusion of mature autophagosome with lysosome [123, 124, 142]. By engaging with the autophagy cargo on defective cellular components (e.g. ubiquitin on damaged mitochondria, more details later), autophagy receptors (e.g. p62/sequestosome) guide expanding autophagosome for selective degradation. The interaction between autophagosomal target (t)-SNARE proteins syntaxin 17 (STX17) [143], synaptosomal-associated protein 29 (SNAP29) and lysosomal vesicle (v)-SNARE protein vesicle-associated membrane protein 8 (VAMP8) [144] are essential for membrane tethering and autophagosome-lysosome fusion.

### Molecular regulation of mitophagy

#### Receptor-mediated mitophagy

**Fig. 8:**
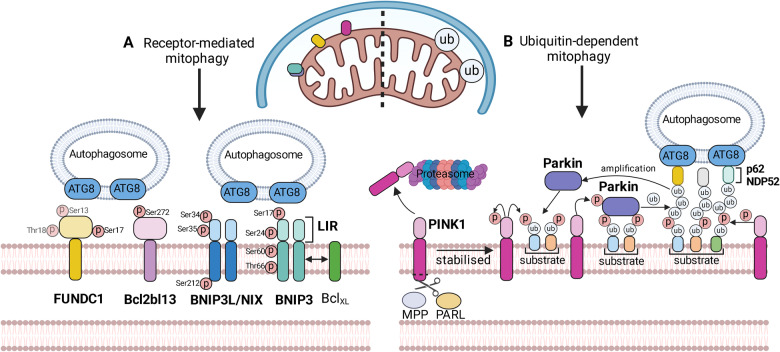
The molecular regulation of mitophagy. Mitophagy can be broadly divided into (**A**) receptor-mediated mitophagy or (**B**) ubiquitination-dependent mitophagy. **A** BNIP3 and BNIP3L/NIX are cytosolic proteins that translocate to the mitochondrial outer membrane to form homodimers during hypoxia, with the LC3-interacting region (LIR) motif oriented towards the cytoplasm to recruit ATG8/LC3-containing autophagosome to induce mitophagy. The pro-mitophagy activity of BNIP3 and BNIP3L is positively regulated by phosphorylation at the indicated sites. BNIP3 may also heterodimerise with Bcl-_XL_ to promote mitophagy. FUNDC1 can be positively and negatively (faded) regulated by phosphorylation at the indicated sites, through the concerted actions of different kinases and phosphatases. Bcl12L13 is another OMM-localised mitophagy receptor that requires phosphorylation to recruit LC3 and elicit mitophagy. **B** PINK1 undergoes basal degradation at mitochondria. When mitochondria are damaged/depolarised, PINK1 processing by PARL is interrupted and PINK1 becomes stabilised on to the OMM. PINK1 then phosphorylates the OMM-bound ubiquitin molecules to promote Parkin recruitment. PINK1 also phosphorylates Parkin to cause Parkin conformational change and to activate its E3 ubiquitin ligase activity. Parkin ubiquitinates many OMM proteins and stimulates the formation of polyubiquitin chains that are recognised by autophagy adaptor proteins, such as sequestosome 1 (SQSTM1/p62), nuclear dot protein 52 (NDP52/CALCOCO2) and optineurin (OPTN). The ubiquitin-bound adaptor proteins contain a LIR motif to recruit LC3-containing autophagosome for the induction of mitophagy. ATG8 autophagy-related protein 8, Bcl-XL B-cell lymphoma-extra large, Bcl12L13 B-cell lymphoma 2-like protein 13, BNIP3 B-cell lymphoma 2 Interacting Protein 3, BNIP3L/NIX B-cell lymphoma 2 Interacting Protein 3-like, FUNDC1 Fun14-domain containing 1, LC3 microtubule-associated protein 1 light chain 3, LIR LC3-interacting region, NDP52 nuclear dot protein 52, OMM outer mitochondrial membrane, OPTN optineurin, PINK1 phosphatase and tensin/PTEN homologue-indced kinase 1, SQSTM1/p62 sequestosome 1. Created with BioRender.com.

Bcl-2 interacting protein 3 (BNIP3) and Bcl-2 interacting protein 3-like (BNIP3L, or NIX) are the most well-studied mitophagy receptors (Fig. 8). BNIP3 is expressed as an inactive monomer in the cytosol under basal condition. During cellular stress, BNIP3 localises to the outer mitochondrial membrane (OMM and forms homodimers to interact with LC3 and recruit autophagosomes through its LIR domain; the phosphorylation of serine residues 17 and 24 flanking the LIR motif stabilises BNIP3/LC3 interaction and promotes mitophagy [145]. BNIP3L shares 50% sequence homology with BNIP3 [146] such that exogenous BNIP3 can completely restore the mitophagy capacity in BNIP3L/NIX-deficient cells [147]. BNIP3L/NIX similarly requires homo-dimerisation and phosphorylation to engage ATG8-containing autophagosome and activate mitophagy [148]; phosphorylation of serine residues 34 and 35 within the LIR domain [149] and of serine residue 212 at the c-terminal [148] are crucial to BNIP3L/NIX-dependent mitophagy. Both BNIP3 and BNIP3L are highly sensitive to hypoxia, which upregulates their transcription mainly through hypoxia-inducible factor 1-alpha (HIF1-α) and may also involve alternative transcriptional factors (e.g. TP53, FOXO3) induced by other cellular stresses/fluctuations [150–152]. Moreover, hypoxia-specific phosphorylation of BNIP3 at serine 60/threonine 66 residues by c-Jun N-terminal kinases 1/2 (JNK1/2) was reported to stabilise BNIP3 interaction with LC3 and promote mitophagy, highlighting a hypoxia-exclusive posttranslational mechanism on BNIP3-dependent mitophagy [153].

The outer mitochondrial membrane contains intrinsic LC3-receptor proteins to promote mitophagy (Fig. 8). FUN14 domain-containing protein 1 (FUNDC1) is an OMM protein that engages with LC3 through its N-terminal LIR domain to induce mitophagy during hypoxia [154] that may be particularly relevant to hypoxic-ischaemic brain injury, including HIE. Unlike BNIP3 and BNIP3L, phosphorylation of the LIR region (serine 13 and tyrosine 18) was shown to suppress FUNDC1-dependent mitophagy [154, 155], while dephosphorylation of serine 13 and tyrosine 18 during hypoxia stimulated mitophagy. On the other hand, hypoxia-activated ULK1 kinase was shown to phosphorylate FUNDC1 at serine 17 and promote mitophagy by interacting with the mitochondrial chaperone protein Lon [156]. Phosphorylation of the serine 17 residue also enabled the recruitment of mitochondrial fission protein Drp-1 by FUNDC1, thus coupling fission and mitophagy during hypoxia [157]. Bcl2-like protein 13 (Bcl2-L-13) is another OMM-localised protein with pro-mitophagy and pro-fission activities [158] with a functional LIR motif between residues W273A and I276A to facilitate Bcl2-L-13/LC3 interaction and mitophagy upon mitochondrial depolarisation.

#### PINK1/Parkin-mediated ubiquitination pathway

PTEN-induced putative kinase 1 (PINK1) is a serine-threonine kinase synthesised in the cytosol and is targeted to mitochondrial compartment via the translocase of outer membrane (TOM) and translocase of inner membrane (TIM) complexes [159, 160] (Fig. 8). Imported PINK1 is cleaved by the inner membrane proteases mitochondrial processing peptidase (MPP) and presenilin-associated rhomboid-like protease (PARL), before releasing the truncated PINK1 into cytoplasm for proteasome degradation [161–163]. Upon mitochondrial depolarisation, PARL-mediated cleavage is halted and PINK1 is stabilised on the outer mitochondrial membrane to associate with TOMs [161, 164]. PINK1 stabilisation on the depolarised OMM is sufficient and necessary for Parkin recruitment and subsequent mitochondrial clearance [165, 166]. In this regard, mitochondrial membrane depolarisation following hypoxia-ischaemia and subsequent reperfusion serve as a potent trigger for PINK1/PARKIN-mediated mitophagy in the post-H/I brain. PINK1 is activated through autophosphorylation and proceeds to phosphorylate ubiquitin molecules bound to OMM proteins. The phosphorylated ubiquitin (phos-Ub) subsequently recruits Parkin from cytosol to associate with OMM [167]. PINK1 then phosphorylates mitochondria-localised Parkin to unleash its E3 ubiquitin ligase activity [168, 169], to enable Parkin-mediated ubiquitination of OMM proteins and formation of polyubiquitin chains. Parkin also ubiquitinates the autophagy adaptor proteins sequestosome 1 (SQSTM1 or p62) and calcium binding and coiled-coil domain 2 (CALCOCO2, or NDP52) that become associated with the ubiquitinated OMM anchors. These autophagy adaptor proteins contain LIR domain and subsequently recruit ATG8/LC3 autophagosome membranes, to direct the autophagic machineries to poly-ubiquitinated mitochondria.

## The mitochondrial hub of cell injury

The growing body of in vitro and in vivo evidence has highlighted the mitochondrion as the injury hub where different RCD pathways converge during neonatal H/I brain injury. Mitochondrial outer membrane permeabilisation (MOMP), which is coordinated by the pro-apoptotic protein family, and the subsequent cytosolic release of cytochrome C, SMAC/DIABLO and apoptosis-inducing factor (AIF) are well-established mechanisms for intrinsic apoptosis and parthanatos in I/R-challenged brain.

Mitochondrial dysfunction has also been suggested to contribute to necroptotic cell death downstream to RIPK1/RIPK3 signalling [170, 171]. Specifically, RIPK3 was reported to target the mitochondrial metabolic complex pyruvate dehydrogenase complex (PDC) to potentiate ROS synthesis [172], and necroptotic cell death could be attenuated by the mitochondrial permeable ROS scavenger butylated hydroxyanisole (BHA) [171]. On the other hand, elimination of dysfunctional mitochondria and mitochondrial ROS through forced mitophagy did not render cells resistant to necroptosis induced by TNF-α or from RIPK3 dimerisation, thus questioning the necessity of mitochondrial ROS in necroptosis execution [173]. The mitochondria-necroptosis interaction is further complicated by study demonstrating that PINK1/Parkin-dependent mitophagy stimulated necroptosis [174]. Further still, RIPK3 was shown to phosphor-activate the mitochondrial serine/threonine phosphatase phosphoglycerate mutase 5 (PGAM5) to promote dynamin-related protein 1 (Drp1)-dependent mitochondrial fission to predispose cells to necroptotic cell death [175]. Another study concluded PGAM5 to be cytoprotective through enhanced PINK1/Parkin mitophagy [176], and one may speculate that RIPK1/RIPK3 activation could have promoted PGAM5-dependent mitophagy and the exact consequent thereof needs to be further studied. In view of the conflicting findings, in-depth mechanistic studies are needed to elucidate the complex necroptosis-mitochondria interactions during HIE pathogenesis.

Mitochondrial involvement may also be relevant to pyroptosis during neonatal H/I brain injury. In I/R-injured microglia and neurons, mitochondrial ROS [84, 85] and mitochondrial DNA [86] released into the cytosol could directly stimulate the NLRP3 inflammasome to induce capase-1 cleavage, IL-1β/IL-18 maturation and GSDMD processing, to culminate to pyroptotic cell death and propagate a pro-inflammatory milieu. Direct recruitment of NLRP inflammasome to the mitochondrial compartment by mitochondrial antiviral signalling protein (MAVS) is observed during viral infection [177, 178], and it remains to be addressed whether a similar endogenous mechanism is in place in response to a “sterile” I/R injury. Current evidences also support a multifaceted role of mitochondria in ferroptosis [179]. ROS synthesised from mitochondrial electron transport chain (ETC) quickly depletes the antioxidant glutathione (GSH) to aggravate lipid peroxidation, a situation that is exemplified by cysteine-deprivation induced ferroptosis and may be mitigated by inhibiting the mitochondrial ETC/tricyclic acid (TCA) cycle [180]. Within mitochondrion, the anaplerotic reaction of glutaminolysis takes place to convert glutamine into downstream metabolic substrates (e.g. α-ketoglutarate) to feed into TCA to trigger ferroptosis in cysteine-deprived cells [181]. Moreover, mitochondrion plays a unique role in cellular iron homoeostasis and controls the amount of free, labile iron available for lipid peroxidation and ferroptosis. In this regard, cytoplasmic iron is transported into mitochondrial fraction via mitoferrin 1 and 2 for the synthesis of heme and Fe-S cluters, and free irons can be sequestered by mitochondrial ferritin (MtFt) [182].

## Conclusion

Hypoxic-ischaemic encephalopathy in term newborn remains a significant clinical problem in approximately half of those affected even with the advent of therapeutic hypothermia. Elucidating the pathological contribution of different modalities of regulated cell death suggests a large number of molecular pathways that can be therapeutically targeted in HIE, while it is important to appreciate that the “crosstalk” between different RCD may require simultaneous intervention. Given that mitochondrion acts as the central regulator for multiple RCD pathways, mitochondrion-focussed strategies have the potential to be developed as more efficacious therapy options for HIE.
